# 9,9-Bis[4-(2-chloro­eth­oxy)phen­yl]-9*H*-fluorene

**DOI:** 10.1107/S1600536810032046

**Published:** 2010-08-18

**Authors:** Kiramat Shah, Muhammad Raza Shah, Islam Ullah Khan, Seik Weng Ng

**Affiliations:** a H.E.J. Research Institute of Chemistry, International Center for Chemical and Biological Sciences, University of Karachi, Karachi 75270, Pakistan; b Department of Chemistry, Government College University, 54000 Lahore, Pakistan; c Department of Chemistry, University of Malaya, 50603 Kuala Lumpur, Malaysia

## Abstract

The title compound, C_29_H_24_Cl_2_O_2_, a fluorene derivative, features a C atom that is connected to four phenyl­ene rings, two of which are almost coplanar (r.m.s. deviation = 0.035 Å) as they belong to the fluorene system. The other two rings are aligned at angles of 67.5 (5) and 85.5 (5)° with respect to the pair. The O and Cl atoms of the –OCH_2_CH_2_Cl– units adopt a *gauche* conformation [torsion angles = 61.6 (6) and 66.6 (5)°].

## Related literature

For related structures, see: Shah *et al.* (2010*a* ▶,*b* ▶).
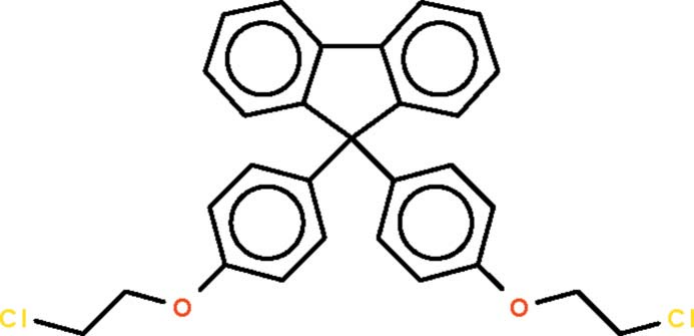


## Experimental

### 

#### Crystal data

C_29_H_24_Cl_2_O_2_
*M* *_r_* = 475.38
Monoclinic, 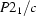

*a* = 12.2334 (7) Å
*b* = 10.8063 (6) Å
*c* = 19.0374 (12) Å
β = 108.172 (2)°
*V* = 2391.2 (2) Å^3^
*Z* = 4
Mo *K*α radiation
μ = 0.30 mm^−1^
*T* = 293 K
0.27 × 0.15 × 0.07 mm

#### Data collection

Bruker Kappa APEXII diffractometer
Absorption correction: multi-scan (*SADABS*; Sheldrick, 1996 ▶) *T*_min_ = 0.783, *T*_max_ = 0.862
18388 measured reflections
4206 independent reflections
2326 reflections with *I* > 2σ(*I*)
*R*_int_ = 0.062

#### Refinement

*R*[*F* ^2^ > 2σ(*F* ^2^)] = 0.061
*wR*(*F* ^2^) = 0.177
*S* = 1.01
4206 reflections
298 parameters
H-atom parameters constrained
Δρ_max_ = 0.53 e Å^−3^
Δρ_min_ = −0.66 e Å^−3^

### 

Data collection: *APEX2* (Bruker, 2009 ▶); cell refinement: *SAINT* (Bruker, 2009 ▶); data reduction: *SAINT*; program(s) used to solve structure: *SHELXS97* (Sheldrick, 2008 ▶); program(s) used to refine structure: *SHELXL97* (Sheldrick, 2008 ▶); molecular graphics: *X-SEED* (Barbour, 2001 ▶); software used to prepare material for publication: *publCIF* (Westrip, 2010 ▶).

## Supplementary Material

Crystal structure: contains datablocks global, I. DOI: 10.1107/S1600536810032046/nk2052sup1.cif

Structure factors: contains datablocks I. DOI: 10.1107/S1600536810032046/nk2052Isup2.hkl

Additional supplementary materials:  crystallographic information; 3D view; checkCIF report

## Figures and Tables

**Fig. 1.**
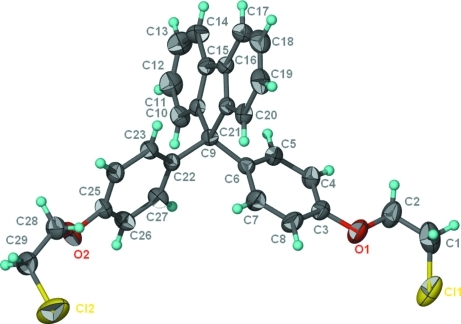
Thermal ellipsoid plot (Barbour, 2001) of C29H24Cl2O2 at the 50% probability level; hydrogen atoms are drawn as spheres of arbitrary radius.

## supplementary crystallographic information

### Comment

We are interested in *V*-shaped molecules; in the title compound, the kink is provided by the carbon atom of the fluorene system that is also connected to two other aromatic rings having *para* substituents (Scheme I). The compound is synthesized from 9,9-bis[4-(2-hydroxyethoxy)phenyl]fluorene, a commerically available reagent. The compound features a carbon atom that is connected to four phenylene rings, two of which are coplanar as these belong the the fluorene system (Fig. 1). The other two rings are aligned at angles of 67.5 (5) and 85.5 (5) ° with respect to the pair. The oxygen and chlorine atoms of the –OCH_2_CH_2_Cl– units adopt a *gauche* conformation [torsion angles 61.6 (6). 66.6 (5) °].

### Experimental

9,9-Bis[4-(2-hydroxyethoxy)phenyl]fluorene (0.5 g, 3.5 mmol) was dissolved in dichloromethane (20 ml) to give a clear solution.Thionyl chloride (5 ml) along with two drops of *N*,*N*-dimethylformamide (to serve as catalyst) were added. The mixture was heated for 12 h. Aqueous ammonium hydroxide was added and the precipitated product was was extracted with dichloromethane. The compound was recrystallized from dichloromethane/hexane (7:3).

### Refinement

Carbon-bound H-atoms were placed in calculated positions (C–H 0.93 to 0.97 Å) and were included in the refinement in the riding model approximation, with *U*(H) set to 1.2*U*(C).

### Crystal data

**Table:**

C_29_H_24_Cl_2_O_2_	*F*(000) = 992
*M**_r_* = 475.38	*D*_x_ = 1.321 Mg m^−^^3^
Monoclinic, *P*2_1_/*c*	Mo *K*α radiation, λ = 0.71073 Å
Hall symbol: -P 2ybc	Cell parameters from 2267 reflections
*a* = 12.2334 (7) Å	θ = 2.3–21.1°
*b* = 10.8063 (6) Å	µ = 0.30 mm^−^^1^
*c* = 19.0374 (12) Å	*T* = 293 K
β = 108.172 (2)°	Wedge, colourless
*V* = 2391.2 (2) Å^3^	0.27 × 0.15 × 0.07 mm
*Z* = 4	

### Data collection

**Table:**

Bruker Kappa APEXII diffractometer	4206 independent reflections
Radiation source: fine-focus sealed tube	2326 reflections with *I* > 2σ(*I*)
graphite	*R*_int_ = 0.062
φ and ω scans	θ_max_ = 25.0°, θ_min_ = 1.8°
Absorption correction: multi-scan (*SADABS*; Sheldrick, 1996)	*h* = −14→10
*T*_min_ = 0.783, *T*_max_ = 0.862	*k* = −12→11
18388 measured reflections	*l* = −22→22

### Refinement

**Table:**

Refinement on *F*^2^	Primary atom site location: structure-invariant direct methods
Least-squares matrix: full	Secondary atom site location: difference Fourier map
*R*[*F*^2^ > 2σ(*F*^2^)] = 0.061	Hydrogen site location: inferred from neighbouring sites
*wR*(*F*^2^) = 0.177	H-atom parameters constrained
*S* = 1.01	*w* = 1/[σ^2^(*F*_o_^2^) + (0.0687*P*)^2^ + 1.9561*P*] where *P* = (*F*_o_^2^ + 2*F*_c_^2^)/3
4206 reflections	(Δ/σ)_max_ = 0.001
298 parameters	Δρ_max_ = 0.53 e Å^−^^3^
0 restraints	Δρ_min_ = −0.66 e Å^−^^3^

### Fractional atomic coordinates and isotropic or equivalent isotropic displacement parameters (Å^2^)

**Table:**

	*x*	*y*	*z*	*U*_iso_*/*U*_eq_	
Cl1	0.99333 (10)	0.53514 (15)	0.63783 (7)	0.0941 (5)	
Cl2	0.08864 (16)	−0.1721 (2)	0.48114 (9)	0.1541 (9)	
O1	0.7730 (2)	0.4956 (3)	0.50972 (15)	0.0763 (9)	
O2	0.1404 (2)	−0.0587 (2)	0.34783 (15)	0.0596 (7)	
C1	0.9155 (5)	0.6374 (5)	0.5696 (3)	0.0966 (18)	
H1A	0.8605	0.6808	0.5879	0.116*	
H1B	0.9681	0.6984	0.5611	0.116*	
C2	0.8516 (4)	0.5776 (5)	0.4969 (2)	0.0719 (13)	
H2A	0.8121	0.6400	0.4614	0.086*	
H2B	0.9049	0.5340	0.4771	0.086*	
C3	0.6879 (3)	0.4493 (3)	0.4503 (2)	0.0510 (10)	
C4	0.6872 (3)	0.4561 (3)	0.3780 (2)	0.0489 (9)	
H4	0.7507	0.4867	0.3667	0.059*	
C5	0.5904 (3)	0.4167 (3)	0.32219 (19)	0.0414 (9)	
H5	0.5901	0.4216	0.2733	0.050*	
C6	0.4953 (3)	0.3708 (3)	0.33692 (18)	0.0372 (8)	
C7	0.5013 (3)	0.3592 (4)	0.4106 (2)	0.0570 (11)	
H7	0.4396	0.3251	0.4226	0.068*	
C8	0.5967 (4)	0.3971 (4)	0.4659 (2)	0.0638 (12)	
H8	0.5993	0.3870	0.5149	0.077*	
C9	0.3808 (3)	0.3497 (3)	0.27615 (17)	0.0355 (8)	
C10	0.3941 (3)	0.3372 (3)	0.19901 (18)	0.0373 (8)	
C11	0.4475 (3)	0.2442 (3)	0.1722 (2)	0.0506 (10)	
H11	0.4818	0.1778	0.2020	0.061*	
C12	0.4487 (4)	0.2521 (4)	0.0999 (2)	0.0658 (12)	
H12	0.4834	0.1895	0.0808	0.079*	
C13	0.3997 (4)	0.3504 (4)	0.0559 (2)	0.0713 (13)	
H13	0.4015	0.3535	0.0074	0.086*	
C14	0.3478 (4)	0.4450 (4)	0.0825 (2)	0.0609 (11)	
H14	0.3150	0.5121	0.0528	0.073*	
C15	0.3458 (3)	0.4375 (3)	0.15460 (19)	0.0426 (9)	
C16	0.2981 (3)	0.5234 (3)	0.19711 (19)	0.0411 (8)	
C17	0.2481 (3)	0.6392 (4)	0.1796 (2)	0.0570 (11)	
H17	0.2381	0.6741	0.1334	0.068*	
C18	0.2135 (3)	0.7017 (4)	0.2324 (3)	0.0632 (12)	
H18	0.1802	0.7795	0.2213	0.076*	
C19	0.2272 (3)	0.6516 (3)	0.3004 (3)	0.0585 (11)	
H19	0.2022	0.6950	0.3347	0.070*	
C20	0.2779 (3)	0.5368 (3)	0.3186 (2)	0.0454 (9)	
H20	0.2872	0.5026	0.3650	0.054*	
C21	0.3148 (3)	0.4731 (3)	0.26698 (18)	0.0373 (8)	
C22	0.3132 (3)	0.2404 (3)	0.29312 (18)	0.0359 (8)	
C23	0.1966 (3)	0.2469 (3)	0.2822 (2)	0.0454 (9)	
H23	0.1576	0.3194	0.2634	0.054*	
C24	0.1356 (3)	0.1487 (3)	0.2986 (2)	0.0494 (10)	
H24	0.0568	0.1554	0.2901	0.059*	
C25	0.1925 (3)	0.0417 (3)	0.32734 (19)	0.0444 (9)	
C26	0.3081 (3)	0.0317 (3)	0.3370 (2)	0.0495 (10)	
H26	0.3467	−0.0414	0.3552	0.059*	
C27	0.3669 (3)	0.1297 (3)	0.3198 (2)	0.0466 (9)	
H27	0.4451	0.1213	0.3262	0.056*	
C28	0.0307 (3)	−0.0406 (4)	0.3561 (2)	0.0630 (11)	
H28A	−0.0259	−0.0266	0.3081	0.076*	
H28B	0.0320	0.0314	0.3868	0.076*	
C29	−0.0001 (4)	−0.1531 (4)	0.3912 (2)	0.0735 (13)	
H29A	0.0058	−0.2254	0.3624	0.088*	
H29B	−0.0793	−0.1464	0.3910	0.088*	

### Atomic displacement parameters (Å^2^)

**Table:**

	*U*^11^	*U*^22^	*U*^33^	*U*^12^	*U*^13^	*U*^23^
Cl1	0.0619 (8)	0.1571 (14)	0.0604 (8)	−0.0277 (8)	0.0152 (6)	−0.0223 (8)
Cl2	0.1338 (15)	0.225 (2)	0.0792 (11)	−0.0733 (14)	−0.0024 (10)	0.0575 (12)
O1	0.067 (2)	0.102 (2)	0.0507 (18)	−0.0338 (18)	0.0035 (16)	−0.0008 (16)
O2	0.0647 (18)	0.0419 (15)	0.082 (2)	−0.0087 (13)	0.0374 (16)	0.0028 (13)
C1	0.128 (5)	0.091 (4)	0.085 (4)	−0.053 (3)	0.053 (4)	−0.026 (3)
C2	0.057 (3)	0.098 (4)	0.061 (3)	−0.029 (3)	0.019 (2)	−0.014 (2)
C3	0.046 (2)	0.057 (2)	0.046 (2)	−0.0065 (19)	0.008 (2)	−0.0004 (19)
C4	0.040 (2)	0.054 (2)	0.056 (2)	−0.0079 (18)	0.019 (2)	−0.0061 (19)
C5	0.041 (2)	0.045 (2)	0.040 (2)	−0.0015 (17)	0.0148 (18)	−0.0036 (16)
C6	0.041 (2)	0.0345 (18)	0.038 (2)	0.0000 (15)	0.0137 (17)	0.0001 (15)
C7	0.054 (3)	0.075 (3)	0.044 (2)	−0.019 (2)	0.017 (2)	0.003 (2)
C8	0.065 (3)	0.087 (3)	0.036 (2)	−0.018 (2)	0.011 (2)	0.004 (2)
C9	0.0362 (19)	0.0375 (18)	0.0336 (19)	0.0002 (15)	0.0121 (16)	0.0006 (15)
C10	0.038 (2)	0.0393 (19)	0.0350 (19)	−0.0052 (15)	0.0116 (16)	−0.0031 (15)
C11	0.057 (3)	0.045 (2)	0.055 (2)	−0.0002 (18)	0.026 (2)	−0.0043 (18)
C12	0.081 (3)	0.066 (3)	0.061 (3)	−0.002 (2)	0.038 (3)	−0.015 (2)
C13	0.094 (4)	0.086 (3)	0.043 (3)	−0.006 (3)	0.034 (3)	−0.010 (2)
C14	0.073 (3)	0.064 (3)	0.045 (2)	0.000 (2)	0.017 (2)	0.009 (2)
C15	0.042 (2)	0.049 (2)	0.036 (2)	−0.0062 (17)	0.0112 (17)	0.0002 (17)
C16	0.038 (2)	0.039 (2)	0.043 (2)	−0.0014 (16)	0.0068 (17)	0.0015 (16)
C17	0.057 (3)	0.047 (2)	0.060 (3)	0.0026 (19)	0.008 (2)	0.010 (2)
C18	0.055 (3)	0.043 (2)	0.086 (3)	0.0076 (19)	0.015 (2)	−0.001 (2)
C19	0.056 (3)	0.044 (2)	0.079 (3)	0.0018 (19)	0.027 (2)	−0.015 (2)
C20	0.044 (2)	0.046 (2)	0.048 (2)	−0.0053 (17)	0.0168 (18)	−0.0093 (17)
C21	0.0326 (19)	0.0363 (18)	0.042 (2)	−0.0032 (15)	0.0107 (16)	−0.0007 (16)
C22	0.036 (2)	0.0369 (19)	0.0349 (19)	−0.0020 (15)	0.0121 (16)	−0.0024 (15)
C23	0.042 (2)	0.040 (2)	0.052 (2)	−0.0007 (17)	0.0110 (18)	0.0040 (17)
C24	0.037 (2)	0.049 (2)	0.060 (2)	−0.0059 (17)	0.0121 (19)	0.0014 (19)
C25	0.049 (2)	0.037 (2)	0.050 (2)	−0.0071 (17)	0.0215 (19)	−0.0018 (17)
C26	0.053 (3)	0.039 (2)	0.060 (3)	0.0064 (18)	0.023 (2)	0.0060 (18)
C27	0.041 (2)	0.046 (2)	0.058 (2)	0.0055 (17)	0.0242 (19)	0.0028 (18)
C28	0.049 (3)	0.066 (3)	0.072 (3)	−0.015 (2)	0.017 (2)	0.008 (2)
C29	0.064 (3)	0.088 (3)	0.066 (3)	−0.025 (2)	0.017 (2)	0.010 (2)

### Geometric parameters (Å, °)

**Table:**

Cl1—C1	1.745 (6)	C13—C14	1.382 (6)
Cl2—C29	1.730 (5)	C13—H13	0.9300
O1—C3	1.371 (4)	C14—C15	1.382 (5)
O1—C2	1.385 (5)	C14—H14	0.9300
O2—C25	1.374 (4)	C15—C16	1.466 (5)
O2—C28	1.413 (5)	C16—C17	1.387 (5)
C1—C2	1.506 (6)	C16—C21	1.392 (4)
C1—H1A	0.9700	C17—C18	1.381 (6)
C1—H1B	0.9700	C17—H17	0.9300
C2—H2A	0.9700	C18—C19	1.365 (6)
C2—H2B	0.9700	C18—H18	0.9300
C3—C8	1.363 (5)	C19—C20	1.382 (5)
C3—C4	1.376 (5)	C19—H19	0.9300
C4—C5	1.388 (5)	C20—C21	1.386 (5)
C4—H4	0.9300	C20—H20	0.9300
C5—C6	1.372 (5)	C22—C23	1.377 (5)
C5—H5	0.9300	C22—C27	1.383 (5)
C6—C7	1.388 (5)	C23—C24	1.387 (5)
C6—C9	1.531 (5)	C23—H23	0.9300
C7—C8	1.368 (5)	C24—C25	1.373 (5)
C7—H7	0.9300	C24—H24	0.9300
C8—H8	0.9300	C25—C26	1.372 (5)
C9—C10	1.533 (4)	C26—C27	1.377 (5)
C9—C22	1.532 (4)	C26—H26	0.9300
C9—C21	1.541 (4)	C27—H27	0.9300
C10—C11	1.379 (5)	C28—C29	1.492 (5)
C10—C15	1.389 (5)	C28—H28A	0.9700
C11—C12	1.385 (5)	C28—H28B	0.9700
C11—H11	0.9300	C29—H29A	0.9700
C12—C13	1.370 (6)	C29—H29B	0.9700
C12—H12	0.9300		
			
C3—O1—C2	118.6 (3)	C13—C14—H14	120.9
C25—O2—C28	117.5 (3)	C14—C15—C10	120.9 (3)
C2—C1—Cl1	114.8 (4)	C14—C15—C16	130.1 (3)
C2—C1—H1A	108.6	C10—C15—C16	109.0 (3)
Cl1—C1—H1A	108.6	C17—C16—C21	120.2 (3)
C2—C1—H1B	108.6	C17—C16—C15	131.3 (3)
Cl1—C1—H1B	108.6	C21—C16—C15	108.4 (3)
H1A—C1—H1B	107.5	C18—C17—C16	118.6 (4)
O1—C2—C1	107.5 (4)	C18—C17—H17	120.7
O1—C2—H2A	110.2	C16—C17—H17	120.7
C1—C2—H2A	110.2	C19—C18—C17	121.4 (4)
O1—C2—H2B	110.2	C19—C18—H18	119.3
C1—C2—H2B	110.2	C17—C18—H18	119.3
H2A—C2—H2B	108.5	C18—C19—C20	120.5 (4)
C8—C3—O1	115.7 (3)	C18—C19—H19	119.8
C8—C3—C4	119.3 (4)	C20—C19—H19	119.8
O1—C3—C4	124.9 (3)	C19—C20—C21	119.1 (4)
C3—C4—C5	119.2 (3)	C19—C20—H20	120.4
C3—C4—H4	120.4	C21—C20—H20	120.4
C5—C4—H4	120.4	C20—C21—C16	120.1 (3)
C6—C5—C4	122.0 (3)	C20—C21—C9	128.6 (3)
C6—C5—H5	119.0	C16—C21—C9	111.2 (3)
C4—C5—H5	119.0	C23—C22—C27	116.8 (3)
C5—C6—C7	117.3 (3)	C23—C22—C9	122.1 (3)
C5—C6—C9	122.3 (3)	C27—C22—C9	121.2 (3)
C7—C6—C9	119.9 (3)	C22—C23—C24	122.1 (3)
C8—C7—C6	121.0 (4)	C22—C23—H23	118.9
C8—C7—H7	119.5	C24—C23—H23	118.9
C6—C7—H7	119.5	C25—C24—C23	119.6 (3)
C3—C8—C7	121.0 (4)	C25—C24—H24	120.2
C3—C8—H8	119.5	C23—C24—H24	120.2
C7—C8—H8	119.5	C26—C25—O2	116.7 (3)
C10—C9—C6	113.0 (3)	C26—C25—C24	119.5 (3)
C10—C9—C22	111.1 (3)	O2—C25—C24	123.8 (3)
C6—C9—C22	112.5 (3)	C25—C26—C27	120.1 (3)
C10—C9—C21	100.2 (3)	C25—C26—H26	120.0
C6—C9—C21	106.3 (3)	C27—C26—H26	120.0
C22—C9—C21	113.1 (3)	C26—C27—C22	121.9 (3)
C11—C10—C15	120.3 (3)	C26—C27—H27	119.0
C11—C10—C9	128.5 (3)	C22—C27—H27	119.0
C15—C10—C9	111.2 (3)	O2—C28—C29	108.6 (3)
C12—C11—C10	118.4 (4)	O2—C28—H28A	110.0
C12—C11—H11	120.8	C29—C28—H28A	110.0
C10—C11—H11	120.8	O2—C28—H28B	110.0
C13—C12—C11	121.2 (4)	C29—C28—H28B	110.0
C13—C12—H12	119.4	H28A—C28—H28B	108.4
C11—C12—H12	119.4	C28—C29—Cl2	111.8 (3)
C12—C13—C14	120.8 (4)	C28—C29—H29A	109.3
C12—C13—H13	119.6	Cl2—C29—H29A	109.3
C14—C13—H13	119.6	C28—C29—H29B	109.3
C15—C14—C13	118.3 (4)	Cl2—C29—H29B	109.3
C15—C14—H14	120.9	H29A—C29—H29B	107.9
			
C3—O1—C2—C1	166.2 (4)	C14—C15—C16—C21	178.5 (4)
Cl1—C1—C2—O1	61.3 (5)	C10—C15—C16—C21	−1.7 (4)
C2—O1—C3—C8	−163.0 (4)	C21—C16—C17—C18	−1.3 (5)
C2—O1—C3—C4	14.7 (6)	C15—C16—C17—C18	−178.0 (4)
C8—C3—C4—C5	4.1 (6)	C16—C17—C18—C19	−0.3 (6)
O1—C3—C4—C5	−173.6 (4)	C17—C18—C19—C20	0.9 (6)
C3—C4—C5—C6	−0.2 (5)	C18—C19—C20—C21	0.0 (6)
C4—C5—C6—C7	−3.1 (5)	C19—C20—C21—C16	−1.6 (5)
C4—C5—C6—C9	168.4 (3)	C19—C20—C21—C9	175.2 (3)
C5—C6—C7—C8	2.7 (6)	C17—C16—C21—C20	2.2 (5)
C9—C6—C7—C8	−169.1 (4)	C15—C16—C21—C20	179.6 (3)
O1—C3—C8—C7	173.3 (4)	C17—C16—C21—C9	−175.1 (3)
C4—C3—C8—C7	−4.6 (7)	C15—C16—C21—C9	2.3 (4)
C6—C7—C8—C3	1.1 (7)	C10—C9—C21—C20	−179.0 (3)
C5—C6—C9—C10	21.6 (4)	C6—C9—C21—C20	−61.2 (4)
C7—C6—C9—C10	−167.0 (3)	C22—C9—C21—C20	62.8 (4)
C5—C6—C9—C22	148.4 (3)	C10—C9—C21—C16	−2.0 (3)
C7—C6—C9—C22	−40.2 (4)	C6—C9—C21—C16	115.8 (3)
C5—C6—C9—C21	−87.3 (4)	C22—C9—C21—C16	−120.3 (3)
C7—C6—C9—C21	84.1 (4)	C10—C9—C22—C23	−96.1 (4)
C6—C9—C10—C11	66.9 (4)	C6—C9—C22—C23	136.1 (3)
C22—C9—C10—C11	−60.6 (4)	C21—C9—C22—C23	15.6 (4)
C21—C9—C10—C11	179.6 (3)	C10—C9—C22—C27	83.4 (4)
C6—C9—C10—C15	−111.7 (3)	C6—C9—C22—C27	−44.4 (4)
C22—C9—C10—C15	120.7 (3)	C21—C9—C22—C27	−164.9 (3)
C21—C9—C10—C15	0.9 (3)	C27—C22—C23—C24	1.4 (5)
C15—C10—C11—C12	−1.7 (5)	C9—C22—C23—C24	−179.1 (3)
C9—C10—C11—C12	179.8 (3)	C22—C23—C24—C25	0.8 (6)
C10—C11—C12—C13	0.9 (6)	C28—O2—C25—C26	164.5 (3)
C11—C12—C13—C14	0.2 (7)	C28—O2—C25—C24	−15.4 (5)
C12—C13—C14—C15	−0.5 (7)	C23—C24—C25—C26	−2.4 (5)
C13—C14—C15—C10	−0.3 (6)	C23—C24—C25—O2	177.5 (3)
C13—C14—C15—C16	179.5 (4)	O2—C25—C26—C27	−178.1 (3)
C11—C10—C15—C14	1.4 (5)	C24—C25—C26—C27	1.8 (6)
C9—C10—C15—C14	−179.8 (3)	C25—C26—C27—C22	0.5 (6)
C11—C10—C15—C16	−178.4 (3)	C23—C22—C27—C26	−2.1 (5)
C9—C10—C15—C16	0.4 (4)	C9—C22—C27—C26	178.4 (3)
C14—C15—C16—C17	−4.5 (6)	C25—O2—C28—C29	−169.9 (3)
C10—C15—C16—C17	175.3 (4)	O2—C28—C29—Cl2	66.6 (4)
